# Cytotoxic and apoptotic effects of six herbal plants against the human hepatocarcinoma (HepG2) cell line

**DOI:** 10.1186/1749-8546-6-39

**Published:** 2011-10-31

**Authors:** Sasipawan Machana, Natthida Weerapreeyakul, Sahapat Barusrux, Apiyada Nonpunya, Bungorn Sripanidkulchai, Thaweesak Thitimetharoch

**Affiliations:** 1 Graduate School, Faculty of Pharmaceutical Sciences, Khon Kaen University, Khon Kaen 40002, Thailand; 2 Center for Research and Development of Herbal Health Products, Division of Pharmaceutical Chemistry, Faculty of Pharmaceutical Sciences, Khon Kaen University, Khon Kaen 40002, Thailand; 3 Centre for Research and Development of Medical Diagnostic Laboratories (CMDL), Faculty of Associated Medical Sciences, Khon Kaen University, Khon Kaen 40002, Thailand

## Abstract

**Background:**

Six plants from Thailand were evaluated for their cytotoxicity and apoptosis induction in human hepatocarcinoma (HepG2) as compared to normal African green monkey kidney epithelial cell lines.

**Methods:**

Ethanol-water crude extracts of the six plants were tested with neutral red assay for their cytotoxicity after 24 hours of exposure to the cells. Apoptotic induction was tested in the HepG2 cells with diamidino-2-phenylindole staining. DNA fragmentation, indicative of apoptosis, was analyzed with agarose gel electrophoresis. Alkylation, indicative of DNA damage, was also evaluated *in vitro *by 4-(4'-nitrobenzyl) pyridine assay.

**Results:**

The extract of *Pinus kesiya *showed the highest selectivity (selectivity index = 9.6) and potent cytotoxicity in the HepG2 cell line, with an IC_50 _value of 52.0 ± 5.8 μg/ml (mean ± standard deviation). Extract of *Catimbium speciosum *exerted cytotoxicity with an IC_50 _value of 55.7 ± 8.1 μg/ml. Crude extracts from *Glochidion daltonii*, *Cladogynos orientalis*, *Acorus tatarinowii *and *Amomum villosum *exhibited cytotoxicity with IC_50 _values ranging 100-500 μg/ml. All crude extracts showed different alkylating abilities *in vitro*. Extracts of *P. kesiya, C. speciosum *and *C. orientalis *caused nuclei morphological changes and DNA laddering.

**Conclusion:**

The extracts of *C. speciosum*, *C. orientalis *and *P. kesiya *induced apoptosis. Among the three plants, *P. kesiya *possessed the most robust anticancer activity, with specific selectivity against HepG2 cells.

## Background

Natural products have been used as anticancer agents [1], such as vincristine and vinblastine from *Catharanthus roseus *[2], taxol and docetaxel from *Taxus brevifolia *[3] and camptothecins from *Camptotheca acuminata *[4]. Even vegetables and fruits may help reduce the risk of cancer in humans [5,6].

We selected six Thai plants for this study, namely *Glochidion daltonii*, *Cladogynos orientalis*, *Catimbium speciosum*, *Acorus tatarinowii*, *Amomum villosum *and *Pinus kesiya *which are also native to China. This study investigates the cytotoxicity of these six plants against the human hepatocarcinoma (HepG2) cell line compared to the normal African green monkey kidney epithelial (Vero) cell line.

## Methods

### Chemicals and reagents

Cell culture media, including Dulbecco's modified Eagle's medium (DMEM), foetal bovine serum (FBS) and penicillin and streptomycin, were purchased from GIBCO^® ^(Invitrogen, USA). Acetonitrile (HPLC grade, Fisher Scientific, UK), *ortho-*phosphoric acid (analytical grade; BHD, UK) and ultrapure water from a Milli-Q system (Millipore, USA) were used for the mobile phase preparation. The reference standards, namely gallic acid, chlorogenic acid, catechin, epicatechin, caffeic acid, vanillic acid, vanillin, coumaric acid, ferulic acid and quercetin, were purchased from Sigma-Aldrich (USA). Dimethylsulfoxide (DMSO), ethidium bromide and the fluorescence dye 4',6-diamidino-2-phenylindole (DAPI) were purchased from Sigma-Aldrich (USA). Sodium bicarbonate (NaHCO_3_), neutral red, 4-(4'-nitrobenzyl) pyridine (NBP) and a standard anticancer drug (melphalan) were purchased from Sigma-Aldrich (USA). A FlexiGene DNA kit was purchased from QIAGEN (Germany); agarose (molecular grade) was purchased from Bio-Rad (USA); and a DNA ladder with stain was purchased from SibEnzyme (Russia). All other reagents used in this study were purchased from Sigma-Aldrich (USA).

### Plants

*G. daltonii*, *C. orientalis*, *C. speciosum*, *A. tatarinowii*, *A. villosum *and *P. kesiya*, were collected from Chaiyaphum province, Thailand and authenticated visually according to a taxonomic method by Assistant Professor Thaweesak Thitimetharoch. The vouchers of the specimens (Table 1) were deposited at the herbarium of the Faculty of Pharmaceutical Sciences, Khon Kaen University, Khon Kaen province, Thailand.

**Table 1:** Samples of the selected plants

Plant name	Family	Collection number	Plant habit	Part used
*Glochidion daltonii* (MÜll. Arg.) Kurz	Euphorbiaceae	TT-OC-SK-839	Tree	Woody stem and twig

*Cladogynos orientalis *Zipp. ex Span	Euphorbiaceae	TT-OC-SK-912	Shrub	Leaves

*Catimbium speciosum *(Wendl.) Holtt.	Zingiberaceae	TT-OC-SK-857	Herb	Rhizomes

*Amomum villosum *var. *xanthioides* (Wallich ex Baker) T. L. Wu & S. J. Chen	Zingiberaceae	TT-OC-SK-941	Herb	Pseudostem and leaves

*Acorus tatarinowii *Schott	Acoraceae	TT-OC-SK-849	Herb	Underground stem (rhizomes)

*Pinus kesiya *Royle	Pinaceae	TT-OC-SK-910	Herb	Woody twig

### Extraction

Dried plants were cut and macerated with 50% ethanol and water (1 g:6 ml) for seven days with occasional shaking. The solvent was filtered, distilled *in vacuo *with a rotary evaporator below 40°C, and freeze-dried to obtain the crude extracts. The percent yields of the extracts and their parts used are shown in Table 2.

**Table 2:** Percent yields and cytotoxic activities.

Sample	Part used	Percent yield	IC_50 _(μg/ml) ± standard deviation (SD)		SI^c^
			HepG2	Vero	
Melphalan^a^			37.7 ± 9.8	59.9 ± 3.2	1.6
*Glochidion daltonii*	Woody stem and twig	4.3	109.1 ± 8.8	38.7 ± 10.0	0.7
*Cladogynos orientalis*	Leaves	7.4	402.0 ± 4.3	205.4 ± 5.6	0.5
*Catimbium speciosum*	Rhizomes	2.6	55.7 ± 8.1	19.7 ± 2.5	0.4
*Acorus tatarinowii*	Underground stem (rhizomes),	10.5	364.8 ± 6.6	182.5 ± 2.1	0.5
*Amommum villosum*	Pseudostem and leaves	7.21	119.2 ± 8.6	Inactive^b^	> 4.2
*Pinus kesiya*	Woody twig	2.2	52.0 ± 5.8	Inactive^b^	9.6

Percent yields of the crude extracts from six indigenous plants and their cytotoxic activities after 24-hour treatment in human hepatocarcinoma (HepG2) and normal African green monkey kidney epithelial (Vero) cell lines.

Data are expressed as the means of triplication.

^a ^Standard anticancer drug and positive control.

^b ^IC_50 _> 500 μg/ml is considered to be inactive.

^c ^SI refers to Selectivity Index, SI value > 3 indicating high selectivity [9].

### HPLC analysis

The HPLC fingerprints of the plant crude extracts were determined by an Agilent 1100 series (USA) with a pumping system (G1310A), a manual injector (G1328B) and a variable UV-Vis wavelength detector (G1314A). Chromatographic separation was performed with a HiQ Sil C_18_W reversed-phase column (4.6 mm id ×250 mm) with a 5 μm particle size (KYA TECH, Japan). An isocratic reversed- phase HPLC was performed. The mobile phase consisted of 20% acetonitrile in 80% Milli-Q water, 0.1% H_3_PO_4_. The flow rate of the mobile phase was maintained at 0.7 ml/min throughout the analysis [7]. The detector wavelengths were set at 213 and 280 nm. The reference standards were gallic acid, chlorogenic acid, catechin, epicatechin, caffeic acid, vanillic acid, vanillin, coumaric acid, ferulic acid and quercetin (1 mg/ml in DMSO), and were used to confirm their presence in the extract fraction. The extracts were dissolved in DMSO at a final concentration of 20 mg/ml.

### Cell culture

Human hepatocarcinoma (HepG2) and normal African green monkey kidney epithelial (Vero) cell lines were maintained at the Centre for Research and Development of Medical Diagnostic Laboratories, Khon Kaen University (Thailand). The cell culture medium was Dulbecco's modified Eagle's medium (DMEM) supplemented with 10% foetal bovine serum (FBS), 100 units/ml penicillin and 100 μg/ml streptomycin. The cells were cultured at 37°C under a humidified atmosphere containing 5% CO_2_.

### Cytotoxic activity

The crude extracts were dissolved in dimethyl sulfoxide (DMSO) at 20 mg/ml as stock solutions which were then diluted with DMEM to desired concentrations ranging from 10 to 500 μg/ml. The final concentration of DMSO in each sample did not exceed 1% v/v, to keep the cytotoxicity of DMSO at less than 10%. The HepG2 cell line and normal Vero cell line were used as cell models. Cytotoxicity testing was performed with a neutral red (NR) method [8]. Melphalan was used as a standard anticancer drug for comparison with the crude extracts. Briefly, the cells were seeded in 96-well plates (100 μl/well at a density of 3 × 10^5^cells/ml) and treated with various concentrations of the samples for 24 hours. Then, cells were washed twice with 1× PBS and the supernatant was discarded. A total of 100 μl NR solution (50 μg/ml) was added to each well and incubated at 37°C for another hour. NR was then dissolved by 100 μl of 0.33% HCl. Absorbance of NR dye was detected by a dual-wavelength UV spectrometer (Anthos 2010; Biochrom, UK) at 520 nm with a 650 nm reference wavelength. The percentage of cytotoxicity compared to the untreated cells was determined with the equation given below. A plot of % cytotoxicity versus sample concentrations was used to calculate the concentration which showed 50% cytotoxicity (IC_50_).

Cytotoxicity (%)=[100×(Absorbance of untreated group−Absorbance of treated group)]/Absorbance of untreated group

The selectivity index (SI), which indicates the cytotoxic selectivity (*ie *safety) of the crude extract against cancer cells versus normal cells [9], was calculated from the IC_50 _of the crude sample in normal cells versus cancer cells.

### Apoptosis induction assay

#### Nuclear staining with DAPI fluorescent dye

Apoptosis of nuclei was detected by a 4',6-diamidino-2-phenylindole (DAPI) staining assay. DAPI dye is a fluorescent dye that stains the nuclear DNA of a cell and is therefore used to determine the effect of plant extracts on inducing morphological changes in the nuclei of cancer cells undergoing apoptosis [10]. Briefly, the HepG2 and Vero cell lines at 500 μl (1 × 10^6 ^cells/well) were seeded on 24-well plates and incubated until cell growth at log phase for 24 hours. The cells were then treated at 2 × IC_50 _of each plant extract or melphalan (at the highest concentration of 500 μg/ml, 0.1% DMSO) for 24 hours. After treatment, the cells were washed with 1× PBS and then fixed with 50 μl of methanol and water (1:1) under -20°C for ten minutes. The fixed cells were washed and stained with 100 μl (1 μg/ml) of DAPI dye and then incubated at 37°C in a dark room for 30 minutes. The excess dye was then removed and 20 μl of PBS:glycerin (1:1) was added to the mixed cells. The cells undergoing apoptosis, represented by the morphological changes of apoptotic nuclei, were observed and imaged from ten eye views at 40× magnifications under an inverted fluorescence microscope. Percentage of apoptotic cells was calculated as follows:

%apoptoticcells=(amountofapoptoticnuclei∕amountofallcells)×100

#### DNA fragmentation detection assay

DNA fragmentation was used to determine the induction of apoptosis induction by observing the biochemical change [11,12]. Briefly, after cancer cells were treated with 2 × IC_50 _of crude extracts and melphalan for 24 hours, the cells were collected and washed with media. Then cell suspensions were transferred to microcentrifuge tubes (1.5 ml) and centrifuged at 300×*g *(Wisd Laboratory instrument, Germany) for five minutes to collect the cell pellets. The DNA in the cell pellet was extracted with Flexigene DNA Kit (QIAGEN, Germany); 2 μg of DNA was electrophoresed on 2% agarose gel containing 0.1 mg/ml ethidium bromide. After electrophoresis, DNA fragments were analyzed with a UV-illuminated camera (Syngene, UK).

### Alkylating activity assay

The pyridine ring of nitrogen in 4-(4'-nitrobenzyl) pyridine (NBP) was used to test the alkylating ability of the test compounds *in vitro*. The pyridine ring 'nitrogen' in NBP models the DNA 'guanine nitrogen' and undergoes alkylation with the test compounds. The NBP assay was conducted with a slightly modified method described previously [13]. Briefly, the mixture solutions of the plant extracts and melphalan were added to a buffer (pH 4.0) solution and incubated at 70°C for 30 minutes with a solution of NBP in microcentrifuge tubes; they were then immediately mixed thoroughly. The mixture solutions were added (in a 1:1 ratio) to test plates in an ice bath containing absolute ethanol and 0.1 N NaOH. The blue colour of the alkylated product was measured for absorbance at 600 nm with a UV-Vis spectrophotometer.

### Statistical analysis

Data were expressed as mean ± standard deviation (SD, *n *= 3). Statistical differences compared between multiple groups of the treated groups and untreated group were analyzed by one way analysis of variance (ANOVA) and followed by Turkey HSD with IBM SPSS version 17.0 (SPSS Inc., USA). Statistical analysis was considered significant if *P *is less than 0.001 and within the 99.9% confidence interval.

## Results and Discussion

### Chemical identification of plant crude extracts using HPLC

HPLC chromatograms were used as references for quality control in future experiments. Commonly found in these plants, the phenolic compounds and flavonoids were used as markers in our experiments. Pure compounds, namely gallic acid, chlorogenic acid, catechin, epicatechin, caffeic acid, vanillic acid, vanillin, coumaric acid, ferulic acid and quercetin, were used as marker compounds (Additional file 1). To confirm the existence of the markers in the crude extracts, we set the detection wavelengths at 213 nm and 280 nm. Retention times of the same marker compound at the two wavelengths were found to be close. HPLC fingerprints of the crude extracts revealed that the peaks of the polyphenolic compounds and flavonoids occurred at the same retention times as the markers (Additional file 2), indicating that all crude extracts consisted of various types of polyphenolic and flavonoid contents (Table 3).

**Table 3:** Amount of the polyphenolic and flavonoid compounds detected in the crude extracts from six indigenous plants.

Compound (at 213 nm)		Amount detected (mg/g crude extracts)					
		*G. daltonii*	*C. orentalis*	*C. speciosum*	*A. villosum*	*A. tatarinowii*	*P. kesiya*
**1**	Gallic acid	32.6	ND	10.7	14.6	11.1	19.7
**2**	Chlorogenic acid	4.2	53.2	ND	2.5	ND	10.1
**3**	Catechin	ND	59.0	ND	1.4	ND	ND
**4**	Epicatechin	ND	56.6	ND	1.4	ND	ND
**5**	Caffeic acid	ND	ND	ND	ND	ND	5.0
**6**	Vanillic acid	ND	23.1	ND	ND	4.6	ND
**7**	Vanillin	ND	4.1	ND	ND	ND	5.0
**8**	Coumaric acid	ND	4.0	ND	ND	1.3	3.1
**9**	Ferulic acid	ND	ND	ND	1.5	1.4	ND
**10**	Quercetin	ND	114.3	ND	ND	ND	ND

The amount of the compounds in the crude extracts was calculated from the HPLC chromatograms detected at wavelength 213 nm under the condition as described in the Methods section.

ND = not detected.

HPLC analysis was performed under specific conditions at specific wavelengths (*ie *213 and 280 nm) and specific mobile phase/stationary phase systems. Therefore, not all chemical compounds in the crude extract were detected. The detected compounds were those that separable under the HPLC conditions and had optimal absorbance at the wavelengths. HPLC fingerprints indicated only the presence of the standard compounds.

### Cytotoxic effect of the plant crude extracts in HepG2 cells

*In vitro *cytotoxicity test is mainly performed to screen potentially toxic compounds that affect basic cellular functions. This toxicity is measured with cellular damage using NR which is a weak cationic dye that penetrates and accumulates in the lysosomes of living cells [8]. Therefore, NR assay was used to determine the cell viability or, in other words, the toxicity of the test compounds. We found that all the crude extracts and melphalan showed significant cytotoxicity to HepG2 and Vero cells, with different IC_50 _values, when compared to the control (*P*< 0.001, one-way ANOVA) (Table 2). In HepG2 cells, no significant difference in mean cytotoxicity (one-way ANOVA) was observed between melphalan and *C. speciosum *(*P*= 0.003), melphalan and *P. kesiya *(*P*= 0.002), *G. daltonii *and *A. villosum *(*P*= 0.964) as well as *C. speciosum *and *P. kesiya *(*P*= 1). In Vero cells, the mean cytotoxicity between the extracts of *A. villosum *and *P. kesiya *was not significantly different (*P*= 1, one-way ANOVA) (Table 2). Melphalan showed the highest cytotoxicity to HepG2 cells but less selectivity. The extract of *P. kesiya *showed relatively high cytotoxicity. The extracts of *P. kesiya *and *A. villosum *were highly selective to HepG2 cells as compared to normal Vero cells. The extracts of *G. daltonii*, *A. villosum*, *A. tatarinowii *and *C. orientalis *were also cytotoxic to HepG2 cells, with IC_50 _values higher than 100 μg/ml. Extract of *A. villosum *was selective to HepG2 cells.

### Apoptosis induction effect of the plant crude extracts in HepG2 cells

**Figure 1:**
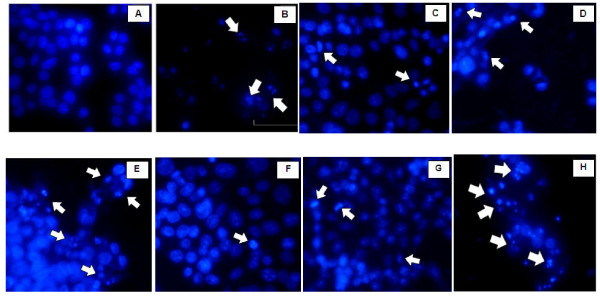
**Nuclear morphological changes**. Nuclear morphological changes in HepG2 cells after treatment with 2 × IC_50 _of crude extracts for 24 hours followed by DAPI staining. (A) Control cells treated with media; (B) melphalan-treated group (80 μg/ml). (C-H) cells treated with extracts of: (C) *G. daltonii *(220 μg/ml); (D) *C. orientalis *(500 μg/ml); (E) *C. speciosum *(110 μg/ml); (F) *A. villosum *(240 μg/ml); (G) *A. tatarinowii *(500 μg/ml); and (H) *Pinus kesiya *(110 μg/ml). Arrows indicate apoptotic bodies of nuclear fragmentation observed at 40× magnification under an inverted fluorescence microscope.

The plant crude extracts were further evaluated for cancer apoptotic death mode. Apoptosis is the pharmacodynamic endpoint of anticancer drug therapy as this phenomenon ensures that no cancer resistance to chemotherapy will occur [11]. Moreover, apoptosis is an autonomous dismantled process to remove individual components of cells and avoids inflammatory effect normally associated with necrosis; thus no toxicity to the normal surrounding cells will occur when cells undergo apoptosis [10,11]. To investigate whether the cytotoxic effects of the crude extracts were due to apoptosis, we treated the HepG2 cells with the plant extracts at 2 × IC_50 _for 24 hours. In the control or untreated HepG2 cells, the stained nuclei were rounded and homogenously stained with DAPI (Figure 1). The treated cancer cells showed different stained DNA nuclei from the control group, by presenting condensed chromatin and apoptotic bodies that are the typical of the early and late stages of apoptosis (Figure 1). According to the statistical analysis of HepG2 cells, melphalan, *C. orientalis*, *C. speciosum*, *A. tatarinowii and P. kesiya *exhibited apoptosis (in percentage) significantly different from the untreated or control group (*P*< 0.001, one-way ANOVA). However, no significant difference of apoptosis was observed (one-way ANOVA) between the extracts of either *A. villosum *(*P*= 0.967) or *G. daltonii *(*P*= 0.003) and the control. The positive control treated with melphalan showed well-separated apoptotic bodies, indicative of the late stage of apoptosis [12] (Figure 1). HepG2 cells treated with 2 × IC_50 _crude extracts from *P. kesiya, C. speciosum *and *C. orientalis *underwent both early and later stages of apoptosis and showed over 30% of apoptotic cells, as observed from nuclear shrinkage, chromatin condensation or apoptotic bodies (after 24 hours of exposure). Extract of *P. kesiya *induced 79.8 ± 8.9% apoptosis in HepG2 cells, which is not significantly different (*P*= 1, one-way ANOVA) from melphalan (80.0 ± 2.8%). Extract of *A. tatarinowii *showed 28.3 ± 3.1% of apoptotic cells mostly in the early state of apoptosis. This early stage of apoptosis was barely observed in the cells treated with the extracts from *G. daltonii *and *A. villosum *which showed less than 10% apoptotic cells (Figure 1). No significant difference was observed (one-way ANOVA) between *G. daltonii *and *A. villosum *(*P*= 0.046), *C. orientalis *and *A. tatarinowii *(*P*= 0.029), *C. orientalis *and *C. speciosum *(*P*= 1) as well as *C. speciosum *and *A. tatarinowii *(*P*= 0.011).

**Figure 2:**
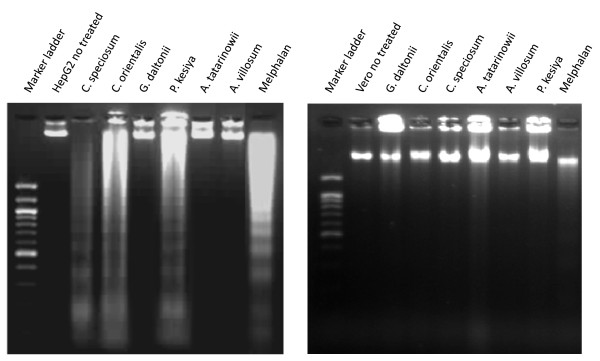
**DNA laddering in (A) HepG2 and (B) Vero cell lines**. DNA laddering was visualized in HepG2 and Vero cell lines after treatment with 2 × IC_50 _of the crude extracts for 24 hours.

Evaluation of apoptosis was further carried out by determining the DNA laddering as a result of DNA fragmentation, indicative of the late stage of apoptosis [12] (Figure 2A). HepG2 cells treated with the extracts of *C. speciosum*, *C. orientalis *and *P. kesiya *showed characteristics of DNA laddering.

Interestingly, the cell samples treated with the extracts of *C. speciosum *and *P. kesiya*, which had apoptotic cells higher than 30%, also showed DNA laddering in HepG2 cells (Table 4). It should be noted that the extract of *P. kesiya *was very selective to HepG2 cells (SI = 9.6) as compared to its cytotoxicity in normal Vero cells. The relatively low cytotoxic extract of *C. orientalis *was also found to cause DNA laddering. The extracts of *G. daltonii*, *A. villosum *and *A. tatarinowii *that possessed low cytotoxicity showed apoptotic cells of less than 30.

**Table 4:** Apoptotic effects of the six plant extracts on HepG2 cells

Plant sample	Percentage of apoptotic cells (mean ± SD)	DNA ladder
Melphalan	80.0 ± 2.8	+
*G. daltonii*	10.1 ± 4.8	-
*C. orientalis*	31.9 ± 3.9	+
*C. speciosum*	36.5 ± 8.1	+
*A. tatarinowii*	28.3 ± 3.1	-
*A. villosum*	3.3 ± 1.2	-
*P. kesiya*	79.8 ± 8.9	+

+ indicates apoptotic DNA ladder; - indicates no apoptotic DNA ladder.

### Alkylating activity of the plant extracts

Results of the NBP assay were used to estimate the mechanism of the DNA damage of many anticancer agents *in vitro *via DNA alkylating activity. The alkylating activities of the plant crude extracts [14] are shown in Table 5. All plant extracts showed significantly different alkylating activity *in vitro *compared to melphalan (*P*< 0.001, one-way ANOVA). Melphalan and the extract of *G. daltonii *showed relatively high alkylating activity, with absorbance higher than 0.5. The extracts of *A. villosum*, *C. orientalis, P. kesiya *and *C. speciosum *showed moderate alkylating activity, with absorbance ranging 0.15-0.5. The *A. tatarinowii *extract showed very low alkylating activity, with an absorbance of 0.135; however, no significant difference of alkylating activity was observed between *C. speciosum *and *A. tatarinowii *(*P*= 0.002, one-way ANOVA).

**Table 5:** Alkylating activities of the six plant extracts and melphalan

Sample	Absorbance (λ_max _= 600 nm)	Alkylation activities
Melphalan	2.768	Absorbance > 0.50 (very high alkylating activities)
*G. daltonii *	0.745	

*A. villosum *var *xanthioides*	0.464	0.15 ≥ Absorbance ≥ 0.50 (high alkylating activities)
*C. orientalis*	0.387	
*P. kesiya *	0.273	
*C. speciosum*	0.176	

*A. tatarinowii*	0.135	Absorbance < 0.15 (low alkylating activities)

It should be noted that this DNA alkylating activity could predict the DNA damage caused by the compound in the cells although it is not the cell-based assay. And since selective anticancer activity in the cancer cells is more preferable, therefore, the DNA fragmentation assay was also conducted in the Vero cells. Results demonstrated that the crude extracts as well as melphalan did not induce DNA fragmentation or apoptosis effect in the normal Vero cells (Figure 2B). The constituents found in the plant crude extracts might alkylate the DNA and cause DNA strand breakage and damage, leading to the cancer cell death. Our finding demonstrated that all crude extracts possessed selective apoptosis induction effect only in the HepG2 cells.

Extract of *C. speciosum *induced high cytotoxicity, apoptosis rates and possessed ability to damage DNA whereas other herbs with more phenolic and flavonoids ingredients had not such anticancer effects. As the UV detector of the HPLC detected the presence of the compounds based on their maximum absorbance at the specific wavelength under specific conditions, not all chemical compounds in the crude extract of *C. speciosum *were detected. This might also be the case for the other herbs as well.

Many studies have suggested that the marker compounds used in our study were the active phytochemicals with anti-cancer, anti-invasive and anti-metastatic activities in cancer cells [15-17]. These phenolic and flavonoid compounds induced apoptosis via the cell cycle arrest progression, increasing pro-protein (Bax and Bad) levels and decreasing anti-apoptotic protein levels (Bcl-2 and Bcl-xL) in the HepG2 cells [15,18-25]. Therefore, the presence of phenolic and flavonoid compounds in the crude extracts was partly attributable to the anticancer activity of the crude extracts.

However, some other active anticancer constituents in the crude extracts may not have been identified under the current HPLC conditions. Extract of *C. speciosum *or *P. kesiya *which contained fewer polyphenols (Table 3) appeared to have strong anticancer activity in the HepG2 cells (Table 2).

## Conclusion

The extracts of *C. speciosum*, *C. orientalis *and *P. kesiya *induced apoptosis. Among the three plants, *P. kesiya *possessed the most robust anticancer activity, with specific selectivity against HepG2 cells.

## Abbreviations

DAPI: 4',6-diamidino-2-phenylindole; DMEM: Dulbecco's modified Eagle's medium; DMSO: dimethyl sulfoxide; FBS: foetal bovine serum; NaOH: sodium hydroxide; NBP: 4-(4'-nitrobenzyl) pyridine; NR: neutral red; HepG2: human hepatocarcinoma cell line; HPLC: high performance liquid chromatography; PBS: phosphate buffer solution; Vero: normal African green monkey kidney epithelial cell line; NCI: National Cancer Institute (USA)

## Competing interests

The authors declare that they have no competing interests.

## Authors' contributions

NW designed the study. SM performed the experiments. TT performed plant authentication. BS carried out plant extraction. SM, AN, NW reviewed the literature and analyzed study results. SB and AN helped draft manuscript and coordinate. SM and NW wrote the manuscript. NW revised the final version of the manuscript. All authors read and approved the final version of the manuscript.

## Supplementary Material

Additional file 1 **HPLC chromatograms of the polyphenolic compounds and flavonoids**. Column: HiQ-Sil C_18_W reversed-phase column; flow rate, 0.7 ml/min. The mobile phase consisted of 20% acetonitrile in 80% Milli-Q water, 0.1% H_3_PO_4 _detected at 213 nm (left) and 280 nm (right). (A) gallic acid; (B) chlorogenic acid; (C) catechin; (D) epicatechin; (E) caffeic acid; (F) vanillic acid; (G) vanillin; (H) coumaric acid; (I) ferulic acid; (J) quercetin; and (K) solvent front or DMSO. Click here for file

Additional file 2 **HPLC fingerprints of the crude extracts**. Column: HiQ-Sil C18W reversed-phase column; flow rate, 0.7 ml per minute. The mobile phase consisted of 20% acetonitrile in 80% Milli-Q water, 0.1% H_3_PO_4 _detected at 213 nm (left) and 280 nm (right). (A) *G. daltonii *extract; (B) *C. orientalis *extract; (C) *C. speciosum *extract; (D) *A. tatarinowii *extract; (E) *A. villosum *extract; and (F) *P. kesiya *extract. Click here for file
