# Supplementary material for: Cytotoxic and apoptotic effects of six herbal plants against the human hepatocarcinoma (HepG2) cell line
Source: Chin Med. 2011 Oct 31;6:39. doi: 10.1186/1749-8546-6-39 (PMC3224580; doi:10.1186/1749-8546-6-39)
Supplement: Additional file 1 — HPLC chromatograms of the polyphenolic compounds and flavonoids. Column: HiQ-Sil C18W reversed-phase column; flow rate, 0.7 ml/min. The mobile phase consisted of 20% acetonitrile in 80% Milli-Q water, 0.1% H3PO4 detected at 213 nm (left) and 280 nm (right). (A) gallic acid; (B) chlorogenic acid; (C) catechin; (D) epicatechin; (E) caffeic acid; (F) vanillic acid; (G) vanillin; (H) coumaric acid; (I) ferulic acid; (J) quercetin; and (K) solvent front or DMSO. [file 1749-8546-6-39-S1.DOC]

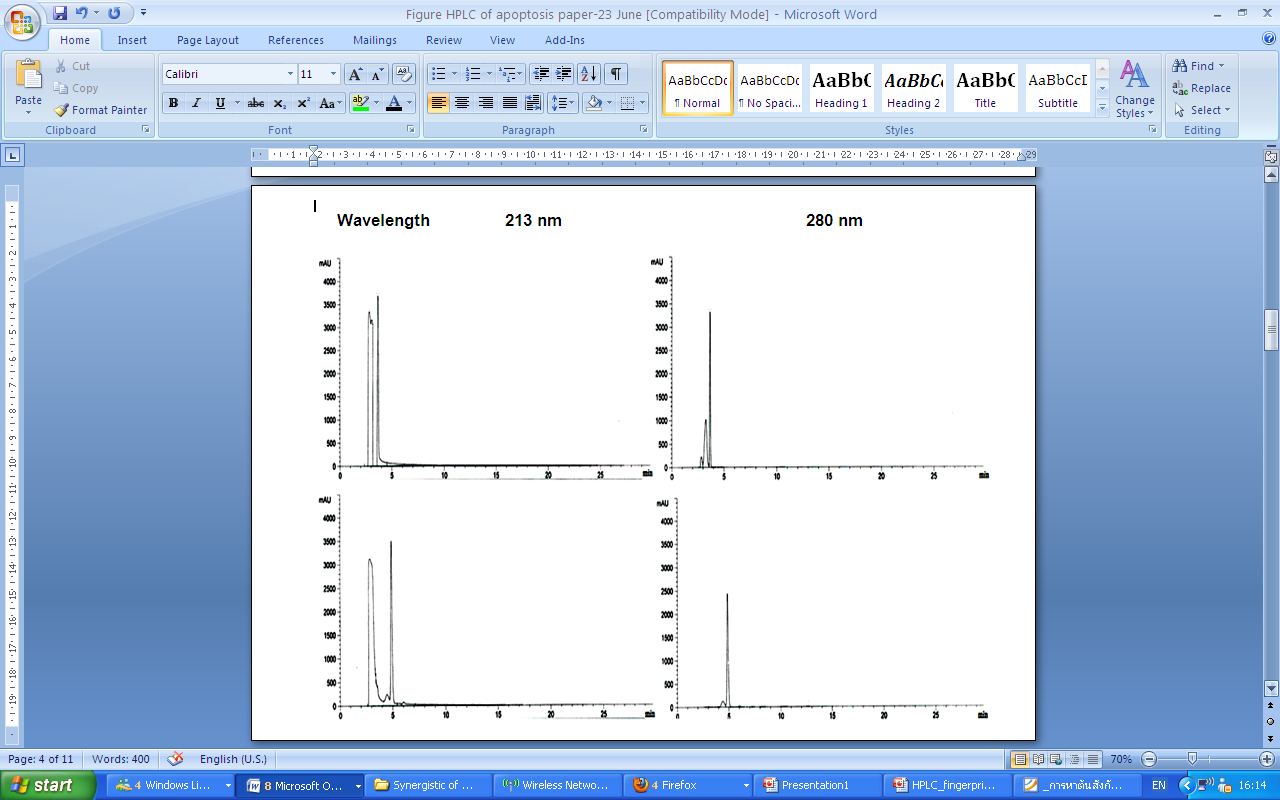


**Wavelength213 nm280 nm**

**B**

**A**

Chlorogenic acid (2)

Gallic acid (1)

Rt = 3.67 min

Rt = 4.87 min

Rt = 3.67 min

Rt = 4.88 min


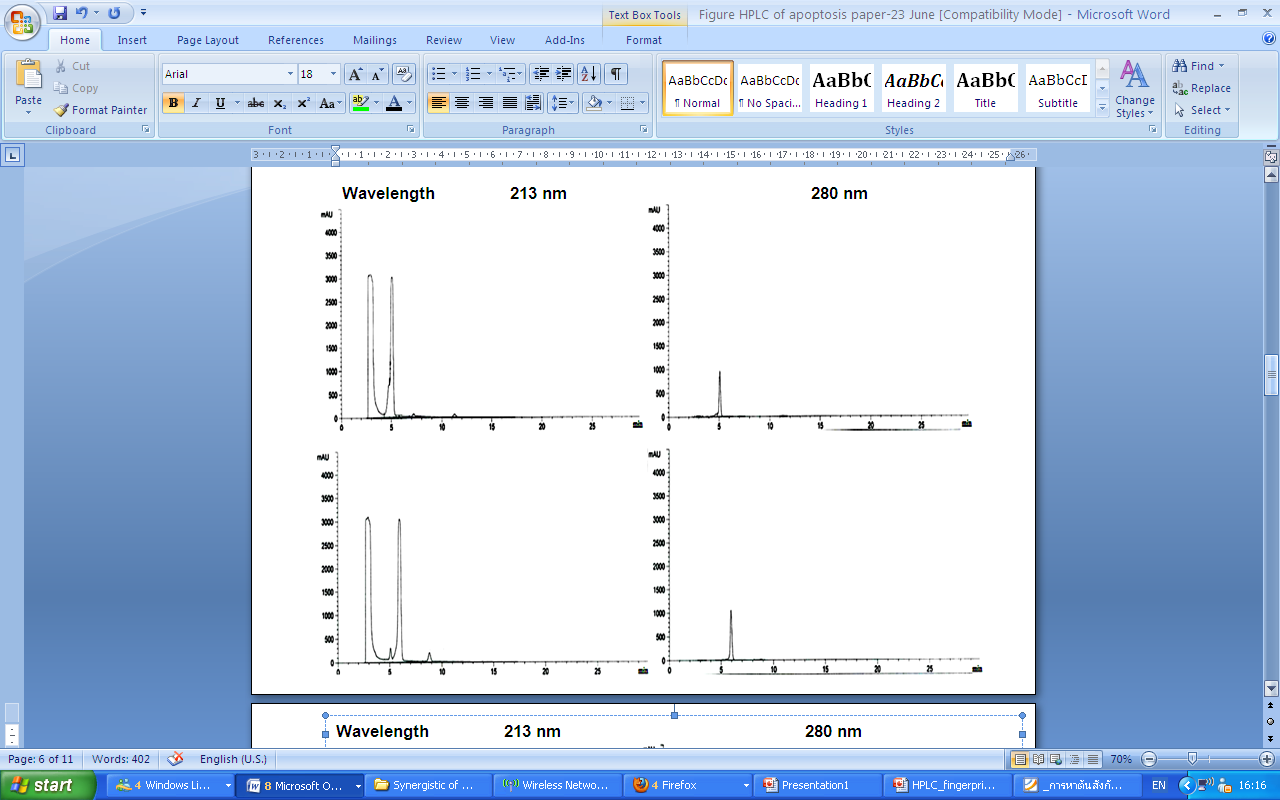

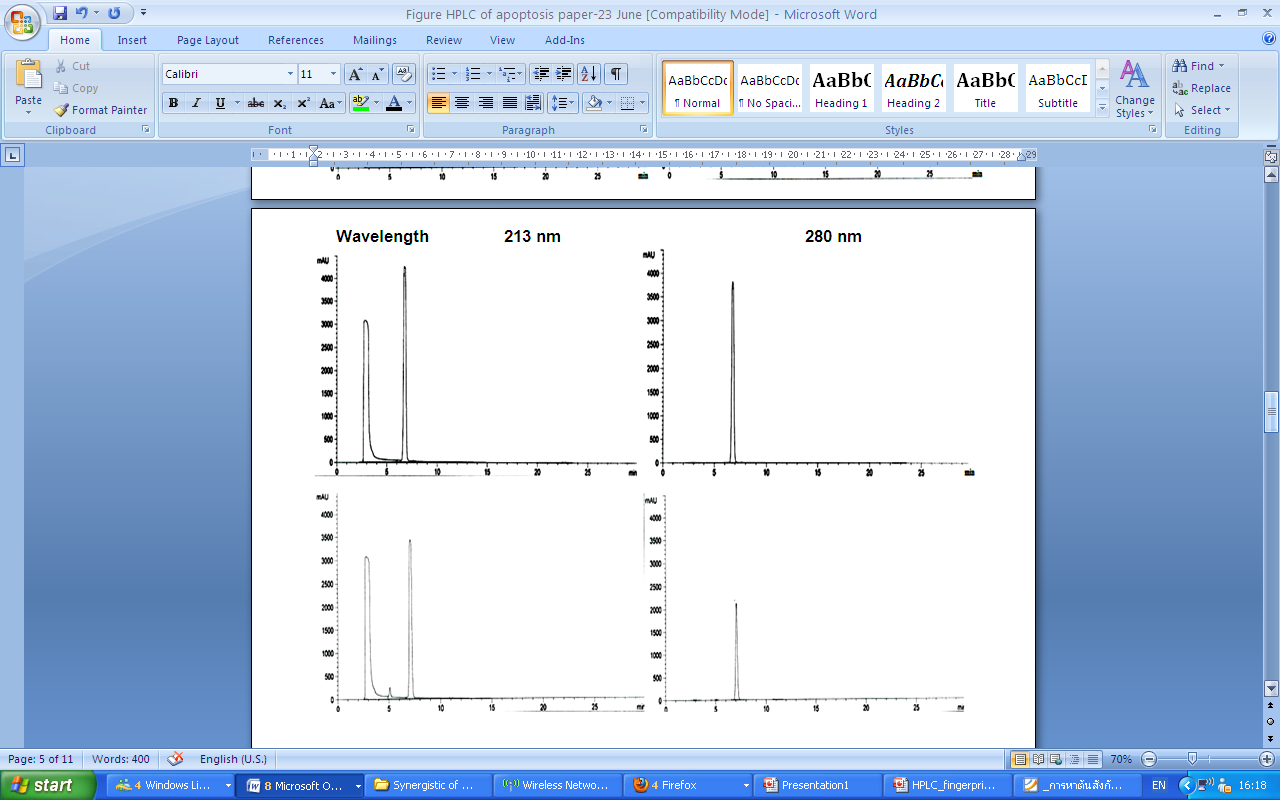

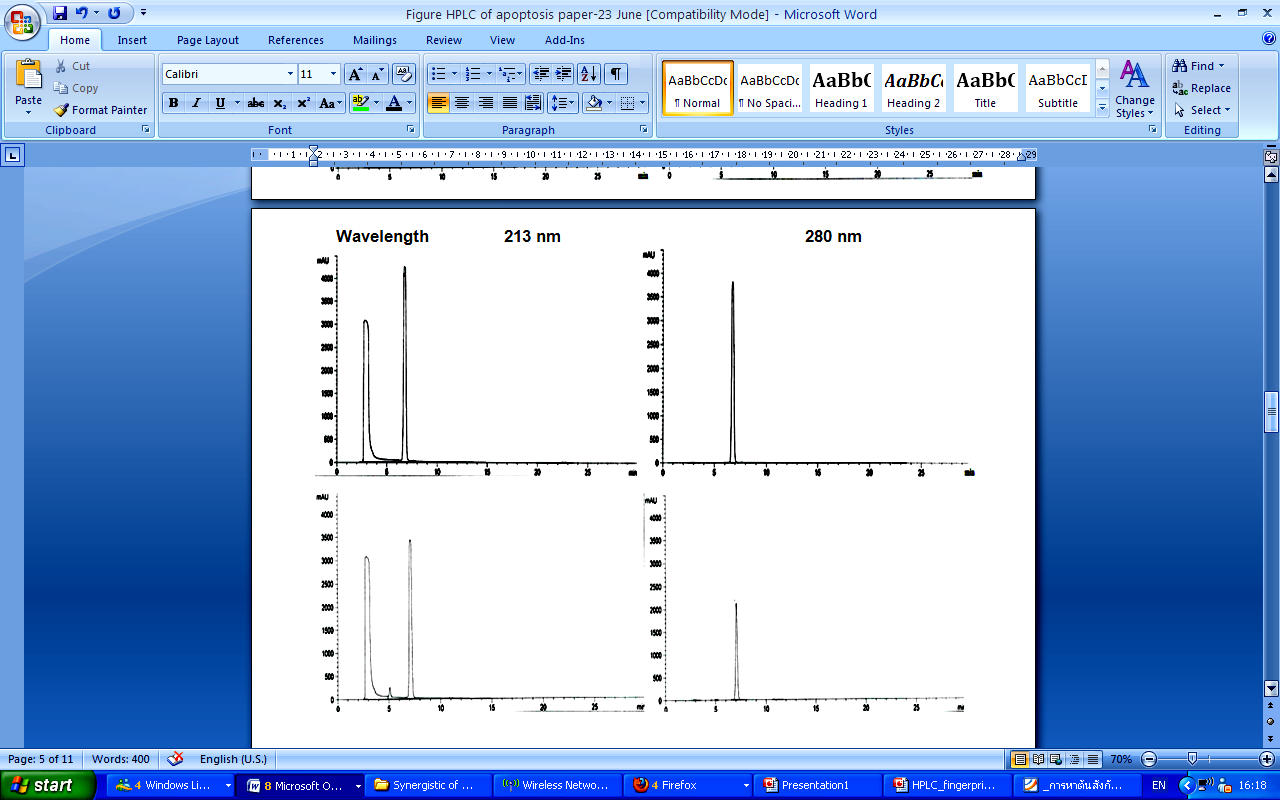


**Wavelength213 nm280 nm**

Epicatechin (4)

Catechin (3)

**D**

**C**

Rt = 5.08 min

Rt = 5.95 min

Rt = 5.07 min

Rt = 5.95 min

**Wavelength213 nm280 nm**

Caffeic acid (5)

Vanillic acid (6)

**E**

**F**

Rt = 6.74 min

Rt = 7.07 min

Rt = 6.74 min

Rt = 7.08 min


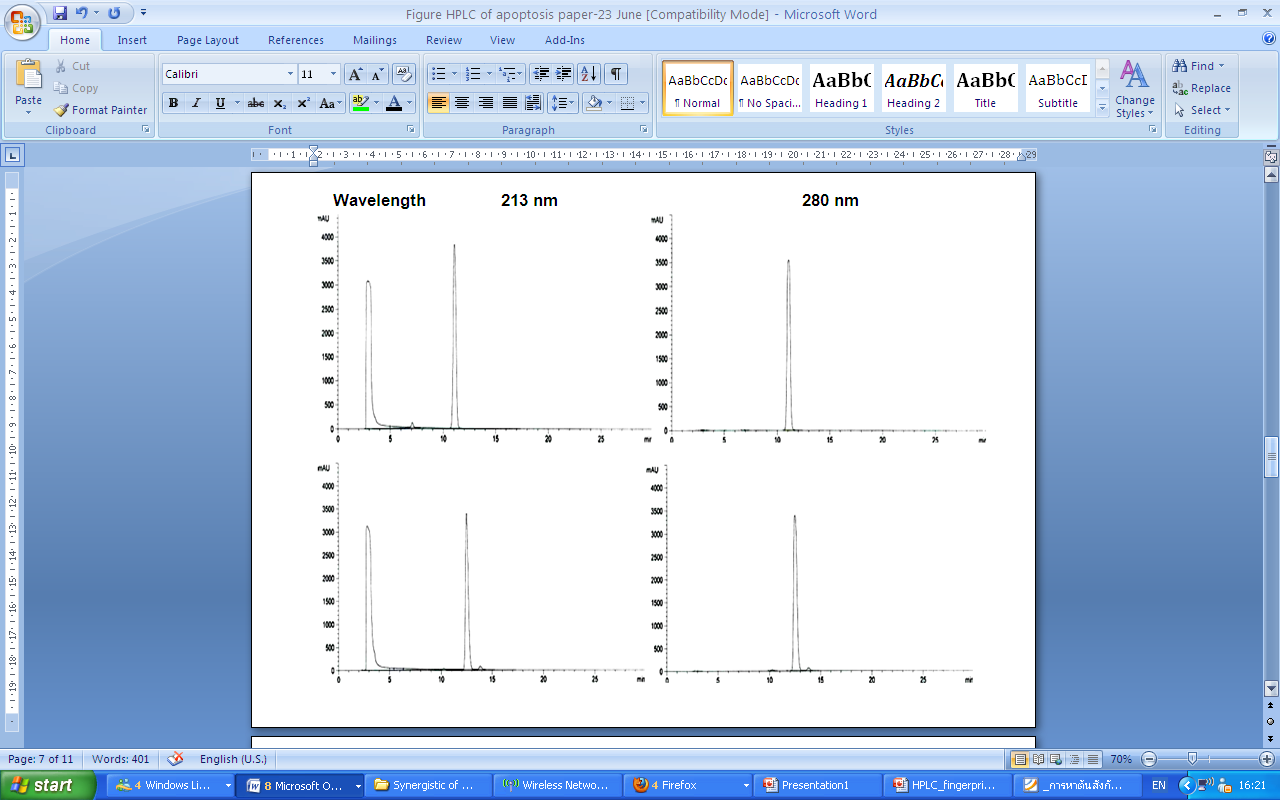

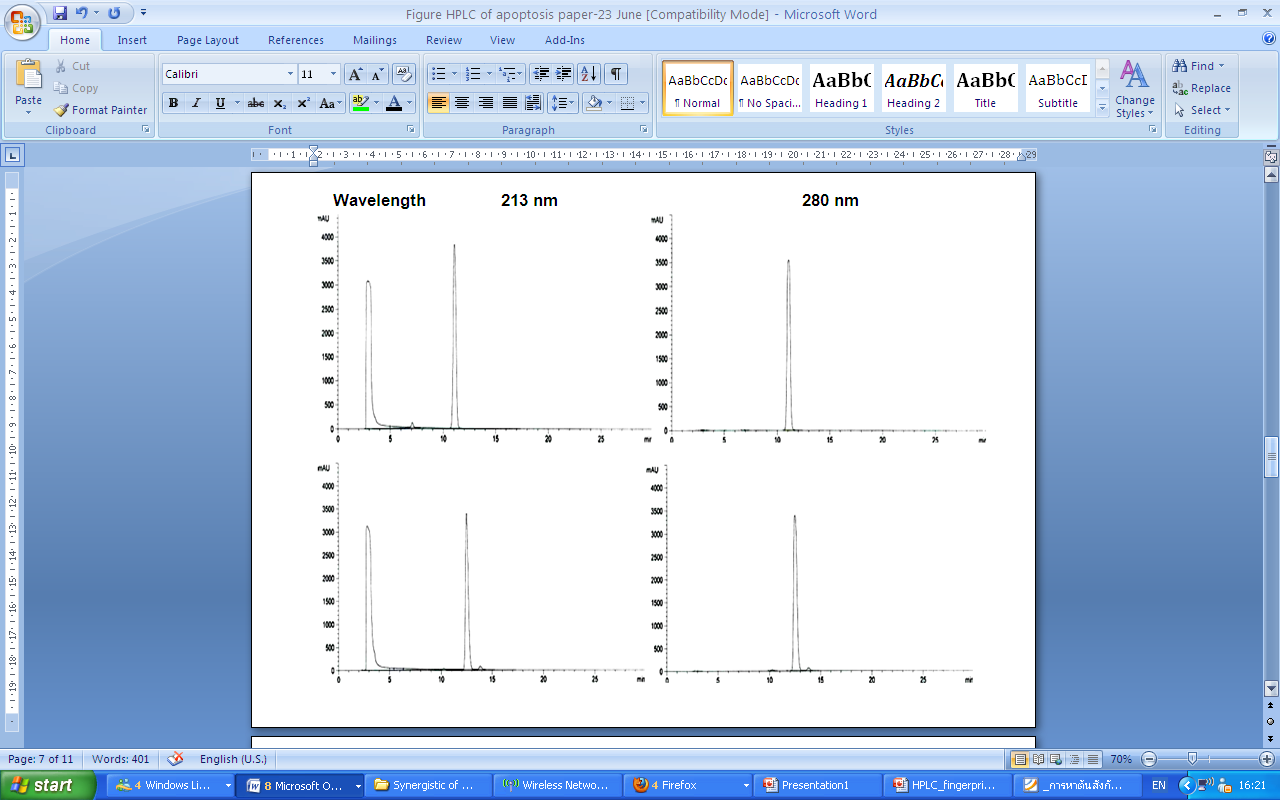


Vanillin (7)

**Wavelength213 nm280 nm**

**G**

**H**

Coumaric acid (8)

Rt = 11.06 min

Rt = 12.47 min

Rt = 11.06 min

Rt = 12.48 min


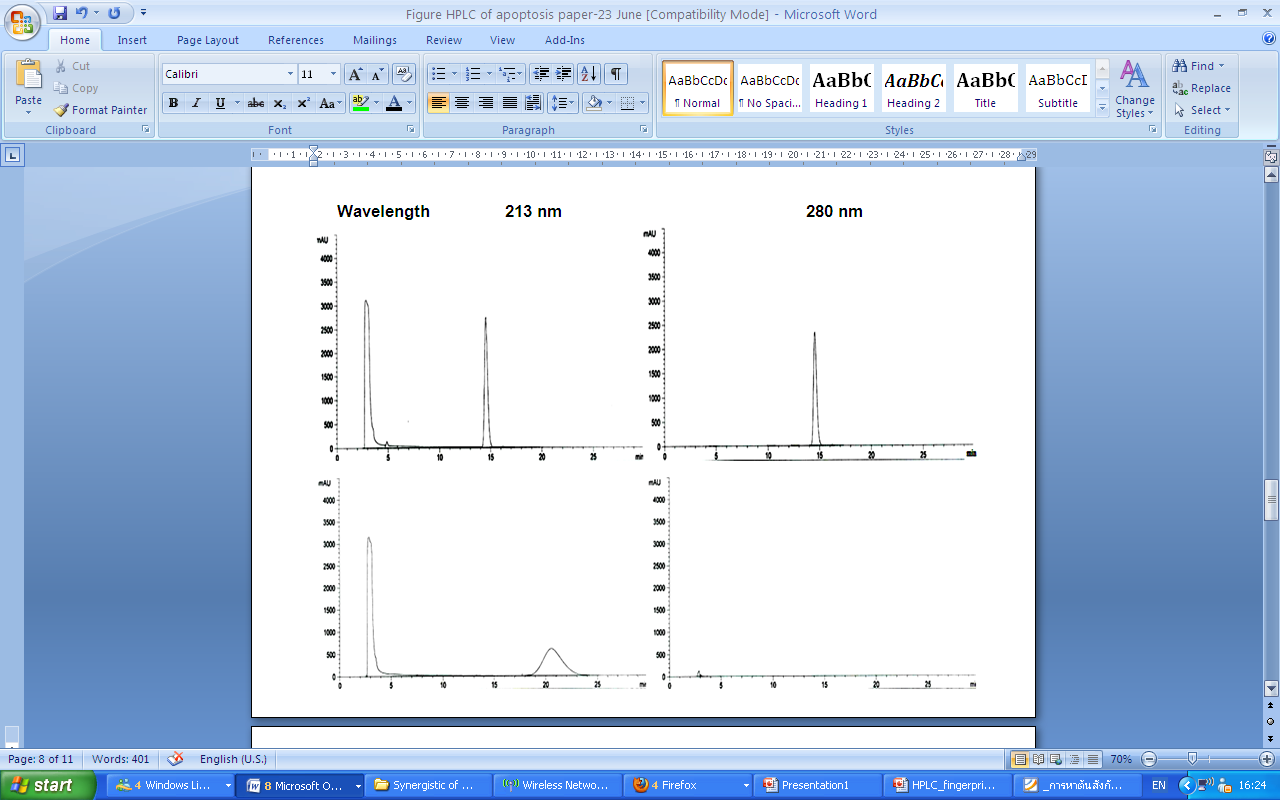

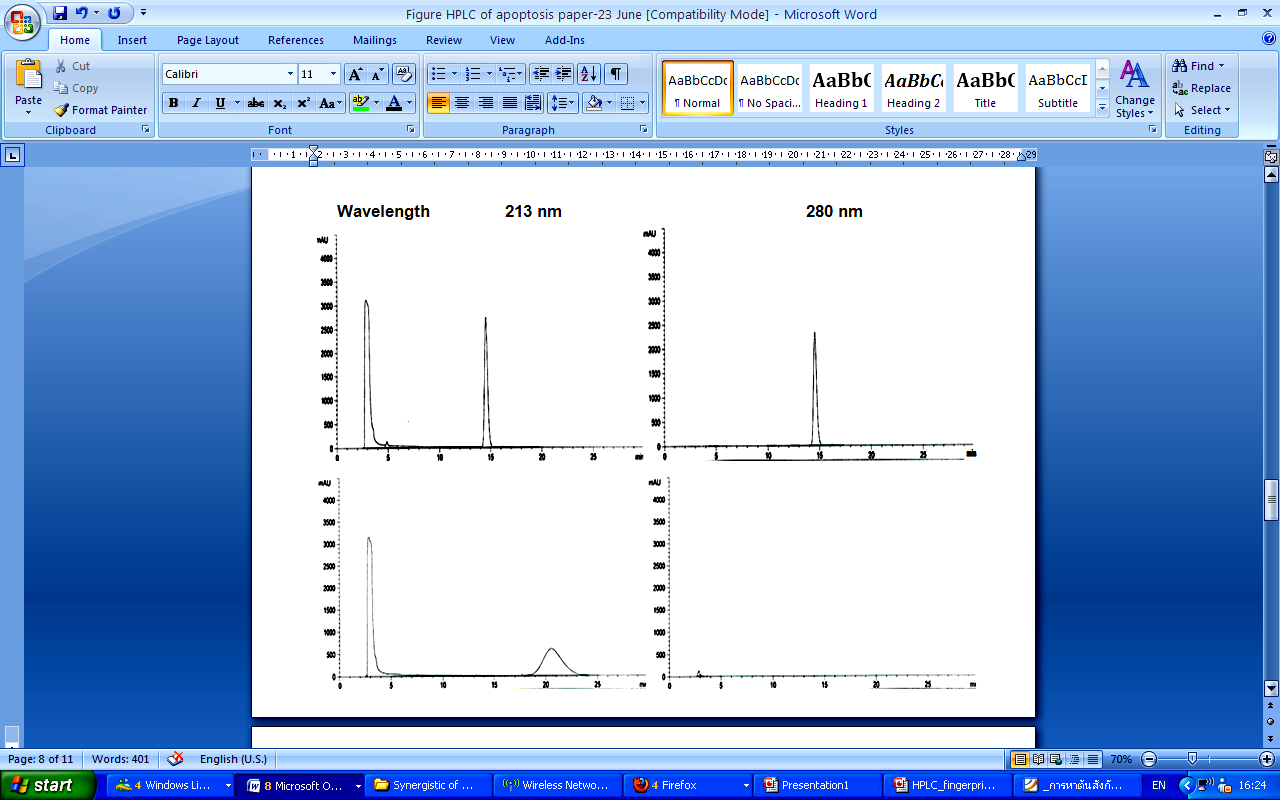


Quercetin (10)

**Wavelength213 nm280 nm**

Ferulic acid (9)

**I**

**J**

Rt = 14.54 min

No peak

Rt = 14.53 min

Rt = 20.55 min


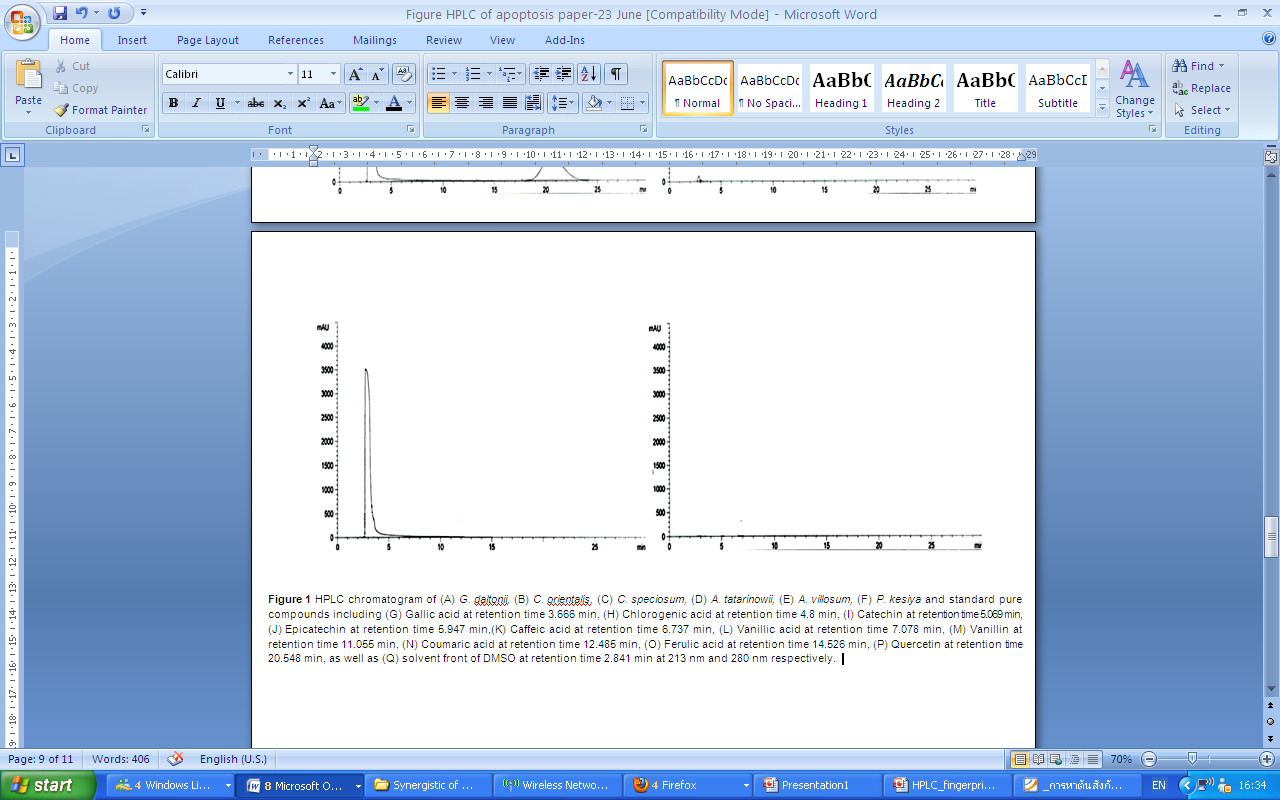


DMSO

**Wavelength213 nm280 nm**

**K**

Rt = 2.841 min

No peak
