# Supplementary material for: Cytotoxic and apoptotic effects of six herbal plants against the human hepatocarcinoma (HepG2) cell line
Source: Chin Med. 2011 Oct 31;6:39. doi: 10.1186/1749-8546-6-39 (PMC3224580; doi:10.1186/1749-8546-6-39)
Supplement: Additional file 2 — HPLC fingerprints of the crude extracts. Column: HiQ-Sil C18W reversed-phase column; flow rate, 0.7 ml per minute. The mobile phase consisted of 20% acetonitrile in 80% Milli-Q water, 0.1% H3PO4 detected at 213 nm (left) and 280 nm (right). (A) G. daltonii extract; (B) C. orientalis extract; (C) C. speciosum extract; (D) A. tatarinowii extract; (E) A. villosum extract; and (F) P. kesiya extract. [file 1749-8546-6-39-S2.DOC]

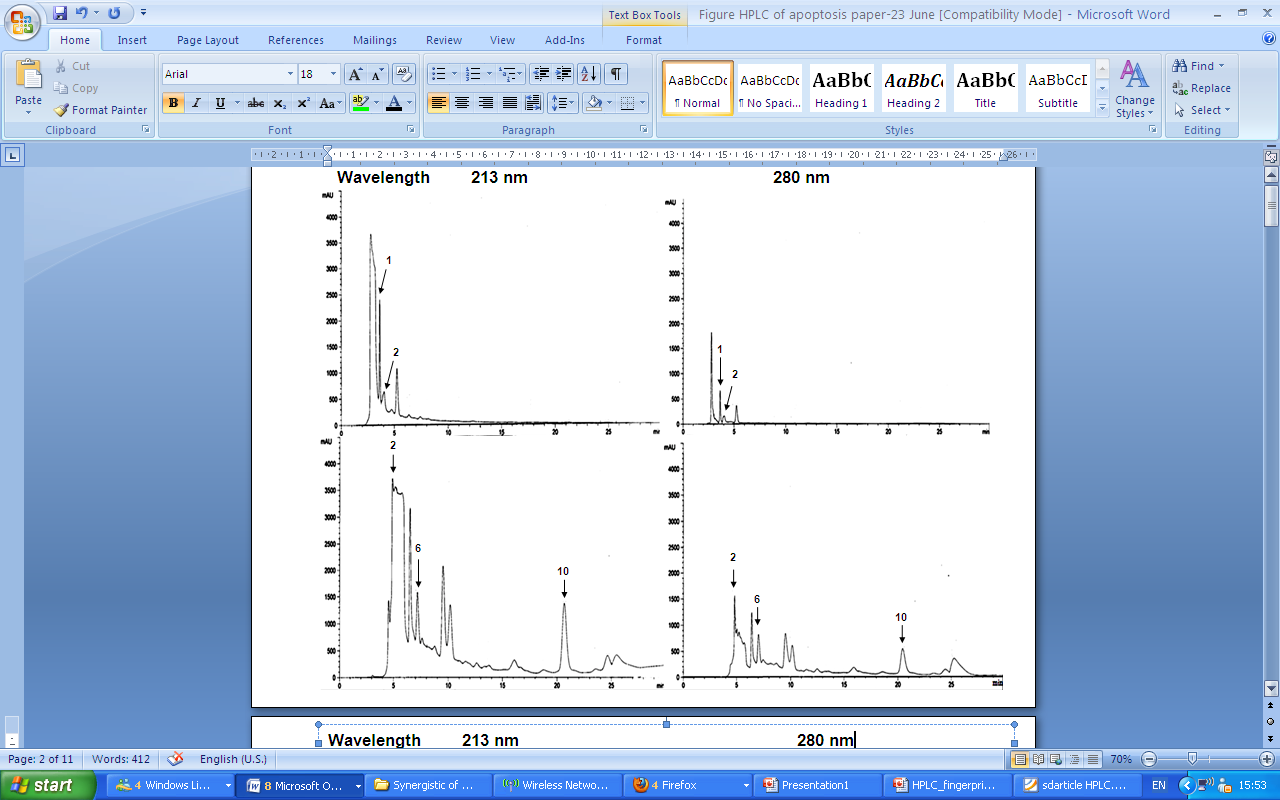

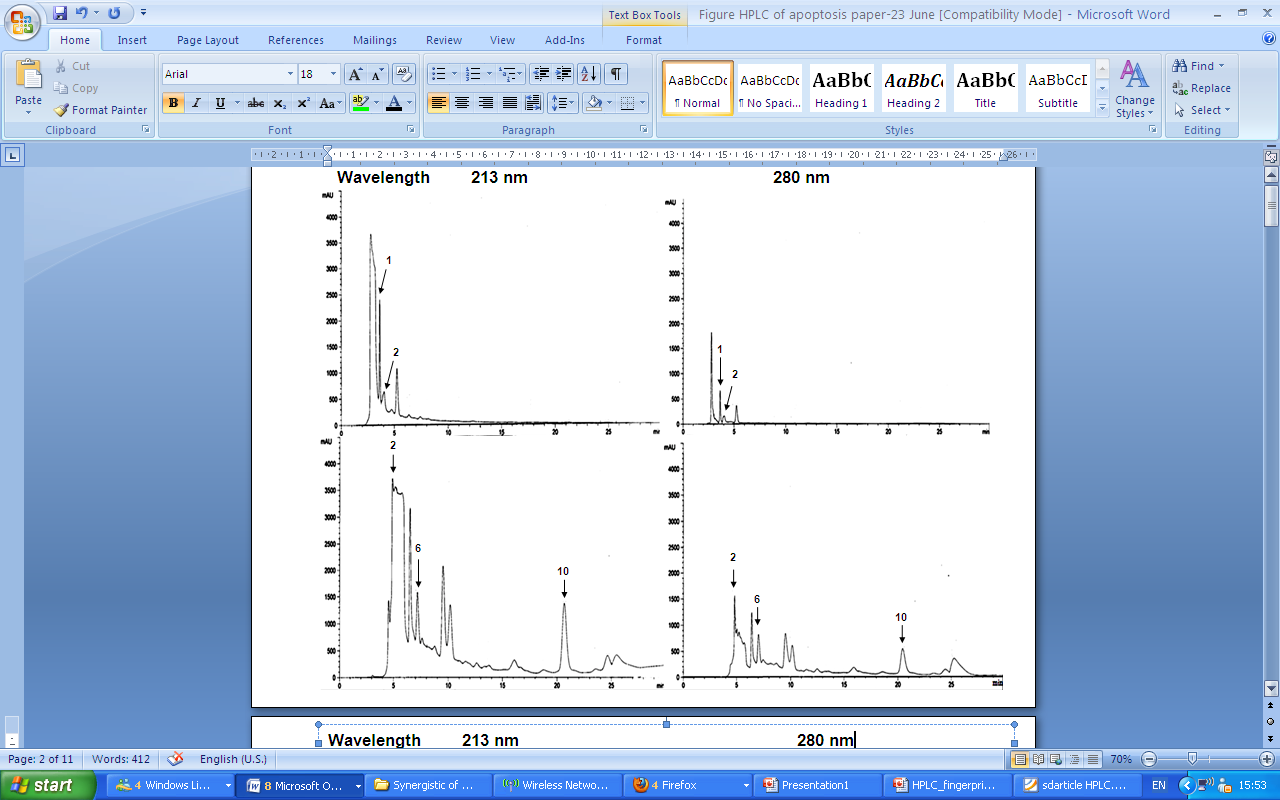


**2**

***G. daltonii***

**B**

**A**

***C. orientalis***

**Wavelength 213 nm 280 nm**


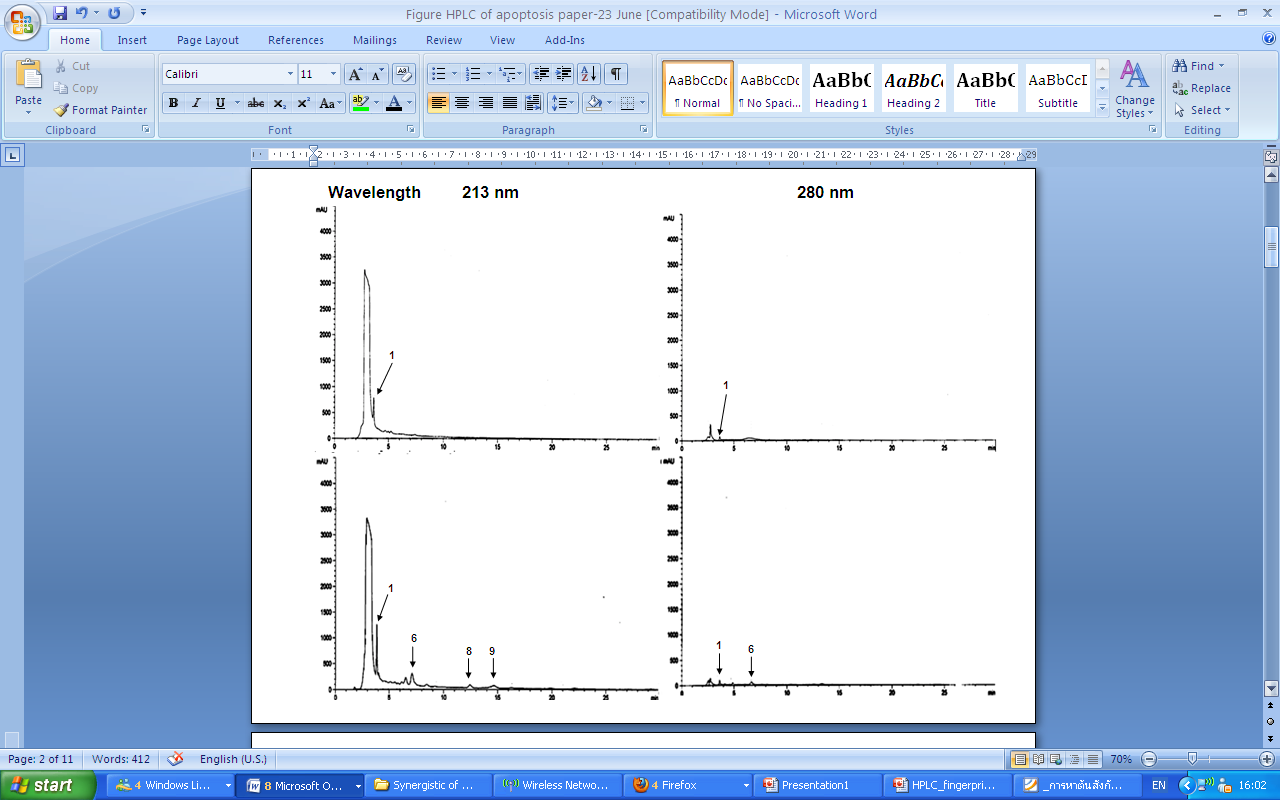

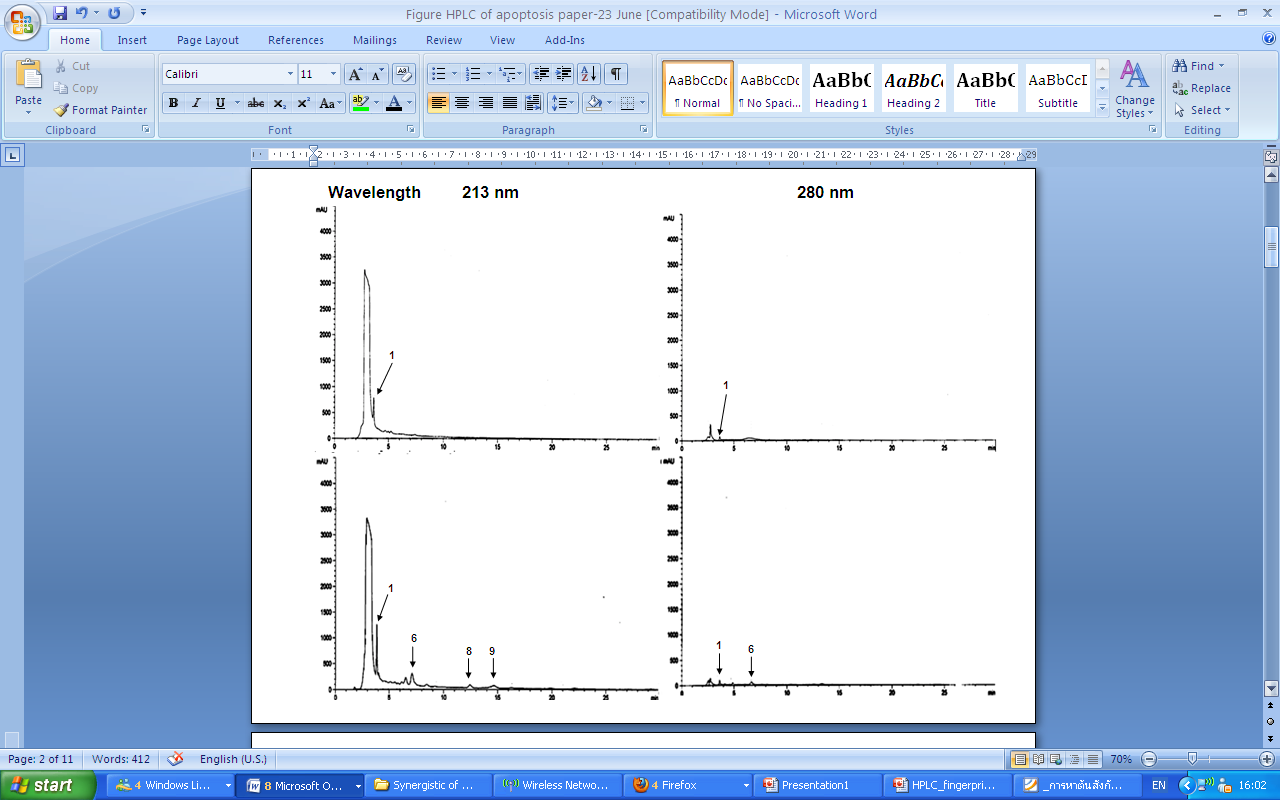


**Wavelength 213 nm 280 nm**

***C. speciosum***

***A. villosum***

**C**

**D**


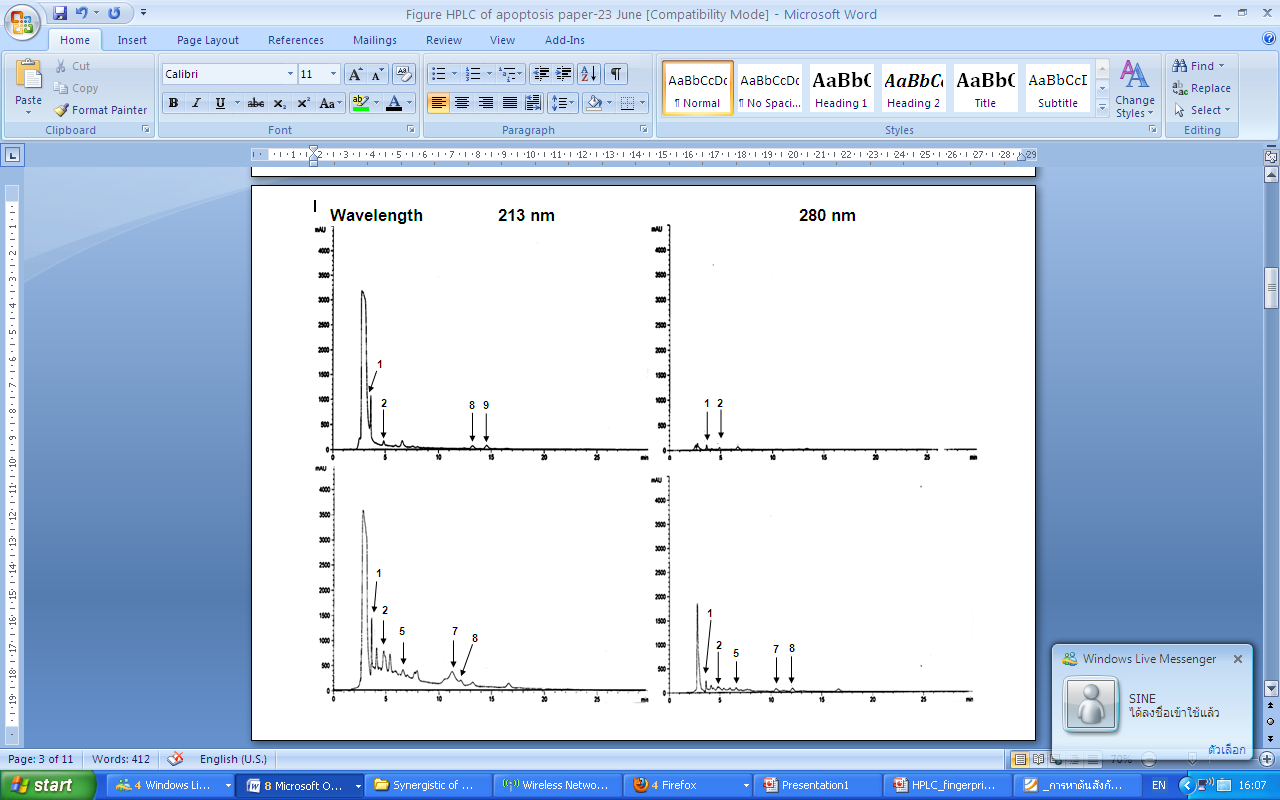

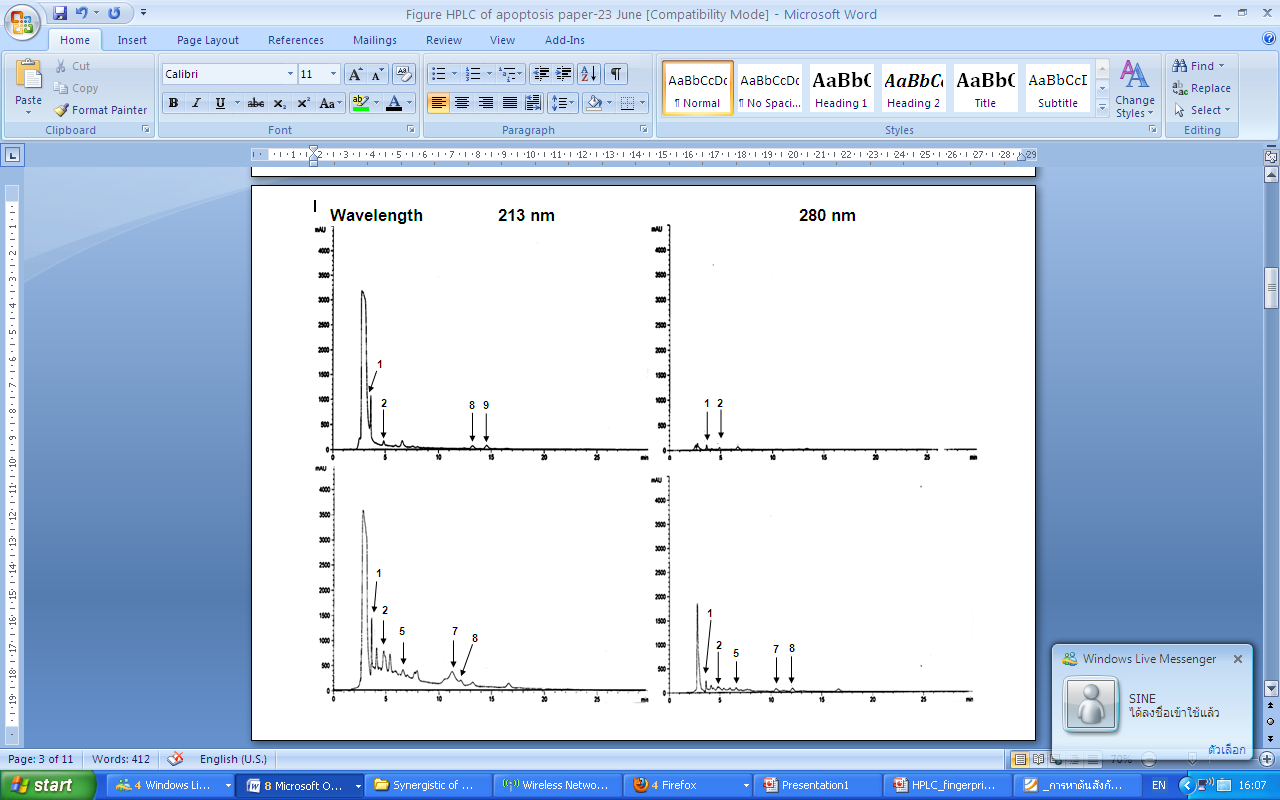


**Wavelength 213 nm 280 nm**

1. ***tatarinowii***

***P. kesiya***

**E**

**F**
